# The Role of Linoleic Acid in Skin and Hair Health: A Review

**DOI:** 10.3390/ijms26010246

**Published:** 2024-12-30

**Authors:** Xi Wang, Yan Jia, Huaming He

**Affiliations:** 1 Beijing Key Laboratory of Plant Resources Research and Development, School of Light Industry Science and Engineering, Beijing Technology and Business University, Beijing 100048, China; wangxi_0304@163.com (X.W.); jiayan@btbu.edu.cn (Y.J.); 2 Institute of Cosmetic Regulatory Science, Beijing Technology and Business University, Beijing 100048, China

**Keywords:** lipid, linoleic acid, skin barrier, hair growth

## Abstract

Lipids are intimately associated with skin condition. This review aims to discuss the function of linoleic acid (LA, 18:2, ω-6), an essential fatty acid, in skin health and hair growth. In skin, LA can be metabolized into ω-6 unsaturated fatty acid, oxidized derivatives and incorporated into complex lipid molecules, including ω-hydroxy-ceramides. Previous research has revealed that skin diseases including acne, atopic dermatitis and psoriasis are associated with disordered LA metabolism. Studies based on animal or skin cell models suggest that LA or LA-rich vegetable oils, topically applied, exhibit diverse biological activities, including the repair of the skin barrier, the promotion of wound healing, skin whitening, photoprotection, anti-inflammatory effects and the stimulation of hair growth. Moreover, the underlying mechanisms of LA’s beneficial effects on skin are summarized. Further research on the correlation of LA metabolism and skin disorders, a deeper exploration of the mechanisms underlying the function of LA in skin management and more investigations of its clinical application are required to enhance the understanding and utilization of LA in cosmetics and pharmaceuticals.

## 1. Introduction

The skin is the largest organ in the human body, functioning as a barrier, in immune defense and in sensation. Skin lipids play a vital role in maintaining the structural and physiological functions of the skin [1]. Fatty acids, which are carboxylic acids with long hydrocarbon chains, perform various physiological roles in the skin, including energy provision, membrane lipid formation, the activation of receptors, their conversion into lipid mediators, and the synthesis of complex lipids [2,3]. The content and composition of fatty acids in the skin significantly influence skin health [4].

Linoleic acid (LA, 18:2, ω-6) is the most abundant polyunsaturated fatty acid (PUFA) in the skin. As an essential fatty acid, LA can only be acquired from the diet or extracutaneous sites and transported across the plasma membrane [5]. LA is abundantly found in various botanical oils, such as safflower, sunflower, corn, soybean, sesame, cottonseed and borage oils [6]. Research [7] has found that compared to hepatocytes and dermal fibroblasts, cultured keratinocytes are more efficient in LA-uptake than oleic acid-uptake, which may help to ensure epidermal capture of sufficient LA. Although the skin, except the sebaceous gland, lacks the enzymes that convert LA to arachidonic acid (AA) [8], LA can be metabolized into oxidative derivatives, or incorporated into complex lipid molecules, e.g., acyl ceramides. Abnormal LA metabolism has been associated with skin diseases, including acne, atopic dermatitis and psoriasis [9,10,11].

As a component of the intercellular lipid matrix in the stratum corneum, LA has been commonly used in cosmetic products such as moisturizers for its moisturizing and skin barrier repair properties [12]. Moreover, the effects of LA or LA-rich vegetable oils on skin and its appendage-hair have been extensively investigated by culture cell models, animal models and clinical trials. A systematic search for studies published before September, 2024 was conducted in the Web of Science, PubMed and Elsevier with the keywords including “linoleic acid”, “skin”, “hair growth”, “metabolism”, “skin barrier” and so forth. This review aims to summarize the metabolic and biological activities of LA in the skin, and explore its potential applications in promoting skin health and hair growth through LA or LA-rich vegetable oils.

## 2. The Metabolism of Linoleic Acid in Skin

### 2.1. Biosynthesis of ω-6 PUFAs

**Figure 1:**
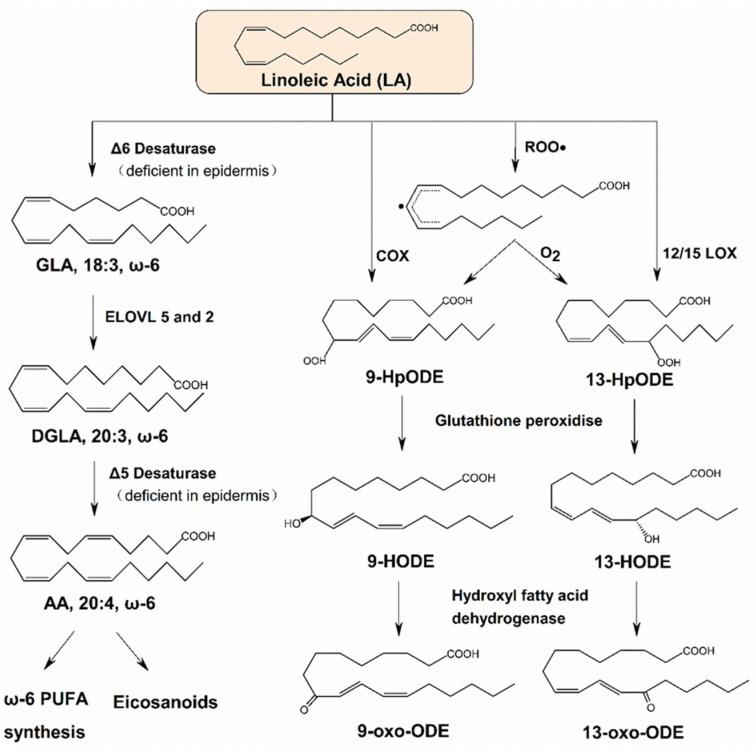
The metabolic pathways of linoleic acid. LA: linoleic acid; GLA: γ-linolenic acid; DGLA: dihomo-γ-linolenic acid; AA: arachidonic acid; 9-HpODE: 9-hydroperoxy-10E, 12Z-octadecadienoic acid; 13-HpODE: 13-hydroperoxy-9Z, 11E-octadecadienoic acid; 9/13-HODE: 9/13hydroxyoctadecadienoic acid; 9/13-oxo-ODE: 9/13-oxo-octadecadienoic acid; ELOVL: elongase of very long chain fatty acids; COX: cyclooxygenase; LOX: lipoxygenase.

Through desaturation and elongation, LA can be converted to other bioactive ω-6 PUFAs, e.g., AA. Subsequently, AA can be metabolized into pro-inflammatory eicosanoids such as prostaglandins and leukotrienes [13] (Figure 1). However, the Δ6 and Δ5 desaturases that catalyze the conversion of LA into AA have been reported to be deficient in the epidermis [8,14] and expressed in differentiating sebocytes in the supra-basal layers of the sebaceous gland [15]. Thus, the skin exhibits limited conversion of LA to AA.

### 2.2. Oxidative Metabolism of Linoleic Acid

In the skin, lipoxygenases (LOXs), particularly 12-LOX and 15-LOX, are the primary enzymes responsible for LA oxidation [16]. Oxidized derivatives of LA include 9/13-hydroxyoctadecadienoic acids (HODEs), 9/13-oxo-octadecadienoic acids (oxo-ODEs), 9/10-epoxy-12-octadecenoate (leukotoxin), and 12/13-epoxy-9-keto-10-trans-octadecenoic acids (EKODEs) [17] (Figure 1).

Among these derivatives, LA-derived 9/13-HODEs are the most abundant hydroxyl fatty acids in human skin, with higher concentrations in the epidermis compared to the dermis [18]. These oxidized derivatives play critical roles in regulating inflammatory processes and cell differentiation [17,19]. In the epidermis, 13-HODE can bind to phosphatidylinositol, forming a novel 13-HODE-containing diacylglycerol (1-acyl-2-13HODE-glycerol), which is pivotal in promoting epidermal cell proliferation and differentiation [20]. Additionally, 13-HODE has been shown to activate NF-κB activity and induce keratin 1 expression, thereby facilitating keratinocyte differentiation [21].

### 2.3. Incorporation of Linoleic Acid into Ceramides

In the skin, free LA is also used to synthesize other lipids, including ceramides (CERs), triglycerides (TGs) and sterols [22,23]. LA is mainly esterified to CER[EOS] (esterified omega-hydroxyacyl-sphingosine), which consists of an omega hydroxylated ultra-long N-chain fatty acid (C28-36) [24]. TG functions as a donor of LA, facilitating its transfer to CER[OS] through the action of PNPLA1-encoded transacylase. This process results in the production of CER[EOS] [25]. CER[EOS] plays a critical role in limiting transdermal water loss and preventing water entry, which is essential for maintaining the integrity of the mammalian epidermal barrier [26].

## 3. Linoleic Acid Metabolism in Skin Diseases

### 3.1. Acne

Acne vulgaris is a chronic inflammatory dermatological condition that affects the pilosebaceous follicles, with a prevalence of approximately 85% among adolescents [27]. An analysis of skin surface lipids found that the proportion of ester-linked LA in CER[EOS] from comedones was only 6%, which was much lower than that in the stratum corneum (41%) [28]. Similarly, a lipidomic-based analysis of skin surface lipids also revealed lower levels of LA in acne patients compared to the non-acne group [29]. The following hypotheses have been proposed to explain the decrease of LA levels in acne patients: (1) Increased production of sebum is essential for acne development. Circulating lipid uptake and β-oxidation are critical steps in sebaceous lipid synthesis. In acne patients, a significant portion of LA may undergo β-oxidation, thus reducing its content in the skin [30]; (2) Acne patients exhibit elevated total sebum secretion, which may dilute the proportion of LA in sebum [11,31]. Despite these hypotheses, the precise mechanisms underlying the reduced LA levels in acne pathogenesis remain unclear and require further investigation.

### 3.2. Atopic Dermatitis

Atopic dermatitis (AD) is one of the most prevalent chronic inflammatory dermatological conditions, with a multifactorial etiology involving both genetic predisposition and environmental triggers [32]. Elevated LA levels in serum have been observed in AD patients, accompanied by reduced levels of its metabolites, such as γ-linolenic acid (GLA) and AA [9,33]. This suggests a potential deficiency in Δ6 desaturase, an enzyme necessary for converting LA to GLA, in AD patients [34]. Furthermore, a negative correlation between trans-epidermal water loss (TEWL) and serum levels of GLA, dihomo-γ-linolenic acid (DGLA) and AA in AD patients indicates a potential relationship between LA metabolites and the maintenance of the epidermal water barrier [9,35]. Together, these results highlight the importance of LA metabolism in maintaining skin hydration and integrity in AD.

### 3.3. Psoriasis

Psoriasis is characterized by chronic immune system activation and the excessive release of pro-inflammatory factors, leading to tissue damage and systemic effects [27,36]. Recently, a lipidomic analysis of psoriatic lesions revealed complex altered levels of LA-containing ceramides and LA-derived oxylipins [24]. In addition, free form LA-derived HODEs including 9-HODE, 13-HODE, 9-oxo-ODE and 13-oxo-ODE were significantly more accumulated in psoriatic skin than in non-psoriatic skin [37]. These results indicate a potential association between LA oxidation metabolism and psoriasis. However, the precise role of LA-derived oxylipins in the progression or resolution of psoriasis remains to be elucidated.

### 3.4. Linoleic Acid in Skin Disease Treatment

So far, several studies have reported the application of LA in skin disease treatment. A clinical trial of patients with mild acne found that the topical application of carbopol gel containing 2.5% LA reduced the size of micro-comedones by approximately 25% after one month of treatment [11]. This suggests LA may function as a comedolytic agent for acne-prone skin. Interestingly, a combination therapy of LA and amitriptyline, a lysosomotropic and anti-apoptotic agent, has been proposed to restore ceramide metabolism, representing a novel topical therapy for AD [38]. A case report further highlighted the efficacy of this combination, which alleviated dryness and itching in patients with mild to moderate AD without causing significant adverse effects [39].

## 4. Functions of Linoleic Acid in Skin and Hair Health

**Figure 2:**
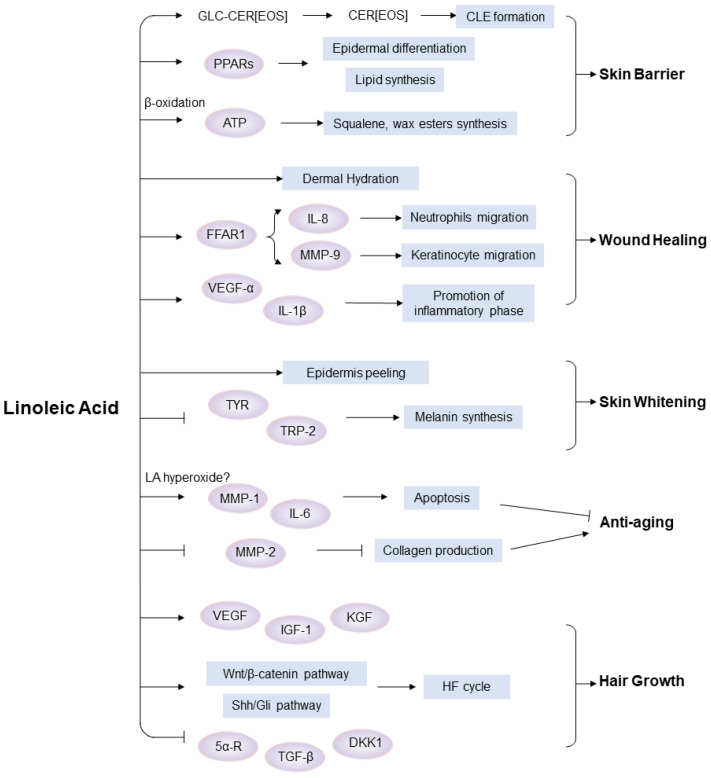
Effects of linoleic acid on skin and hair growth. LA or LA-rich vegetable oils, topically applied, exhibit various effects, including the repair of the skin barrier, the promotion of wound healing, skin whitening, anti-aging and the stimulation of hair growth. LA can enhance the skin barrier via its incorporation into CER[EOS], the regulation of epidermal differentiation and the promotion of lipid production; LA facilitates wound healing via the promotion of skin hydration, enhancement of the migration of neutrophils and keratinocytes and acceleration of the inflammatory process; LA application can promote epidermal peeling, inhibit tyrosinase activity and subsequently melanin synthesis, thereby achieving the purpose of whitening. The anti-aging effects of LA are controversial. LA may induce apoptosis and promote aging, while it may inhibit MMP-2 activity, thereby reducing collagen degradation and achieving anti-aging effects; LA can regulate hair growth-related pathways and growth factors, thereby influencing the hair growth cycle.

A summary of the diverse functions of LA in skin and hair as well as the underlying mechanisms is shown in Figure 2.

### 4.1. Skin Barrier

In rodents, deficiency of essential fatty acids leads to the development of squamous skin and increased skin permeability, both of which can be restored through the topical application of free or esterified LA [40]. This demonstrates the pivotal role of LA in maintaining the integrity of the skin barrier. Consistent with this, a recent study using tissue-engineered skin models showed that supplementation with 10 μM LA in the culture medium enhanced the barrier function of the skin model [41].

LA plays a role in skin barrier maintenance through different mechanisms, with the incorporation of LA into ceramides being the most well-established and well-recognized. Linoleate-containing CER[EOS] is a critical component of the skin’s lipid barrier [26,42]. Recent studies have identified that LA-containing acyl glucosylceramide (GLC-CER[EOS]) generates CER[EOS] under the continuous oxidation of LOX enzymes, including 12R-LOX and eLOX3. A portion of this is further converted to ω-hydroxyacyl-sphingosine (OS) and ω-hydroxy-very long-chain fatty acids (VLFAs), which are covalently attached to the outer surface of the keratinocyte envelope (CE), a structure composed of cross-linked proteins. These covalently bound lipids constitute the corneocyte lipid envelope (CLE) [23,43]. Long-chain acyl-CoA synthase 1 (ACSL1), which is expressed in the epidermis, converts LA into linoleoyl-CoA. In Acs1^−/−^ mice, the content of CER[EOS] containing ω-O-esterified LA in the epidermis was found to be decreased, resulting in an impaired skin barrier [44].

Another mechanism through which LA exerts its influence on the skin barrier is by activating peroxisome proliferator-activated receptors (PPARs) as ligands. PPARs are involved in various pathways, including lipid metabolism, inflammation, keratinocyte differentiation and permeability barrier homeostasis [45]. In an in vitro model of fetal skin development, the application of LA promoted skin barrier formation and significantly reduced trans-epidermal water loss (TEWL) by activating PPAR-α [46]. The lipid-rich sebum produced by sebocytes serves as a protective coating for the skin. Several studies have indicated that LA treatment can stimulate lipogenesis in sebocytes. Specifically, treatment with 10^−4^ mol/L LA was observed to significantly induce lipid accumulation in immortalized human SZ95 sebocytes [47]. Furthermore, a lipidomic analysis revealed more pronounced changes in the lipid profile of sebocytes treated with 1 µM LA compared to those treated with 1 µM AA, suggesting LA as a potent regulator of sebaceous lipogenesis [48]. Additionally, LA can provide energy through β-oxidation, thus facilitating sebaceous glands to synthesize squalene and wax esters, which are associated with the skin barrier [49].

### 4.2. Wound Healing

Wound healing is a complex process that includes inflammation, angiogenesis, cell migration and extracellular matrix synthesis and remodeling and involves many different cell lineages (neutrophils, macrophages, endothelial cells, keratinocytes and fibroblasts) [50]. Upon the formation of a skin wound, the initial response is the formation of a clot. Subsequently, inflammatory cells, including innate immune cells, neutrophils and macrophages, migrate to the wound site to induce the inflammatory response. This is followed by the formation of granulation tissue, which requires angiogenesis, fibroblast migration and extracellular matrix deposition. Finally, epidermal migration and cell division are initiated to restore the integrity of the barrier [51,52].

The topical application of LA has been suggested to promote wound healing. In a clinical trial, the application of LA-containing creams was demonstrated to prevent pressure ulcers in patients. This effect was associated with improved hydration and elasticity, indicating that the maintenance of optimal hydration is a key mechanism through which LA facilitates wound healing [51]. Furthermore, the beneficial impact of LA on wound healing has been corroborated in animal models. The topical administration of 30 μM LA to BALB/c mice resulted in accelerated tissue healing and a significant improvement in wound recovery after 48 h [52]. Similarly, the topical administration of LA was found to enhance wound healing tissue mass in Wistar rats, accompanied by an increased number of neutrophils in the wounded area [53]. A further investigation of cultured neutrophils revealed that the supplementation of LA (5~200 mM) in medium increased the production of vascular endothelial growth factor (VEGF-α) and interleukin-1 beta (IL-1β) in a dose-dependent manner [53]. This suggests that LA accelerates the wound healing process through its pro-inflammatory effect. Additionally, a study has demonstrated that LA-induced migration, matrix metalloproteinase-9 (MMP-9) activity and interleukin-8 (IL-8) expression in HaCaT cells can be mediated through free fatty acid receptor 1 (FFAR1) [54]. The collective findings of these studies suggest that LA facilitates wound healing by modulating the inflammatory response.

Several studies have investigated the effect of LA-rich vegetable oils on the wound healing process [55,56]. Lucuma nut oil (*Pouteria lucuma* (Ruiz and Pav.) Kuntze) containing 38.9% LA was demonstrated to promote the regeneration of endothelial cells in zebrafish at concentration of 20–100 μg/mL. It also induced rapid cutaneous wound closure in CD-1 mice at doses of 500 and 1000 μg [55]. The application of pumpkin seed oil (*Cucurbita pepo* L.) containing 50% LA to the dorsal wound of Wistar rats at a dose of 0.52 μL/mm^2^ every two days reduced bleeding time, stabilized fibrin and promoted the migration of fibroblasts, thereby accelerating wound healing [56]. Thus, LA-rich vegetable oils represent a promising treatment for wound healing.

Overall, the mechanisms by which LA and LA-rich vegetable oils facilitate wound healing encompass the promotion of neutrophil and keratinocyte migration, the induction of pro-inflammatory factors, the stimulation of endothelial cell regeneration and the maintenance of skin hydration.

### 4.3. Skin Whitening

Skin color is largely determined by the quantity of melanin produced by melanocytes. The synthesis of melanin pigments in the melanosome requires various enzymes, among which tyrosinase (TYR), tyrosinase-related protein 1 (TRP1) and tyrosinase-related protein 2 (TRP2) have been identified as rate-limiting enzymes [57].

The skin whitening effect of LA has been evaluated in both cultured cell and animal models. The incubation of B16F10 melanoma cells with 25 μM LA significantly reduced TYR protein levels and decreased melanin synthesis, likely through the accelerated proteolytic degradation of TYR [58]. The topical application of 0.5% LA to the UVB-stimulated hyperpigmented dorsal skin of brownish guinea pigs resulted in an efficient whitening effect. This was attributed to the inhibition of melanin production in melanocytes and the promotion of melanin desquamation pigment from the epidermis [59]. LA-rich vegetable oils also exhibited skin-whitening properties. The treatment of B16F10 melanoma cells with spent coffee ground oil (containing 40% LA), or rubber (*Hevea brasiliensis* (Willd. ex A. Juss.) Müll. Arg.) seed oil (containing 33% LA) was observed to reduce melanin content by inhibiting the activities of TYR and TRP-2 [60,61]. Overall, the mechanisms underlying the function of LA and LA-rich vegetable oils in skin whitening include the promotion of the desquamation of melanin pigment from the epidermis and the inhibition of TYR and TRP-2 activity.

### 4.4. Photoprotection

Ultraviolet (UV) radiation is the major cause of skin cancer and skin aging. Prolonged exposure to UV light can lead to the apoptosis of keratinocytes, induction of inflammation and a reduction in collagen synthesis [12]. Furthermore, UV radiation has been reported to induce oxidative stress in skin cells and higher lipid peroxidation levels of serum have been observed in patients with skin diseases [62].

A previous study reported that LA treatment did not elevate the levels of TNF-α or IL-1α in UVB-irradiated human keratinocytes [63]. Similarly, in UVB-irradiated hairless mice, edema and erythema scores were significantly reduced following the topical application of a LA-containing cream (40%) in comparison to a basal cream [64]. The topical administration of LA-rich ginseng oil (*Panax ginseng* Meyer) (31.48% LA) for a period of 21 days was found to inhibit the incidence of UVC-induced apoptosis in mice. This was evidenced by increased Bcl-2 (anti-apoptotic) expression and reduced Bax (pro-apoptotic) expression, resulting in a decreased Bax/Bcl-2 ratio. Nevertheless, it remains uncertain whether the anti-apoptotic effect is achieved by LA components [65]. Conversely, an in vivo study examining the responses of tape-stripped human skin to UV exposure revealed that the long-time use of LA might aggravate UV-induced damage. The topical application of LA for two days was observed to promote the apoptosis of dermal cells and to increase the expression of MMP-1 and IL-6 mRNA in UV-irradiated areas in comparison to the vehicle [12]. The effect may be attributed to the induction of LA hyperoxides following UV irradiation or to the inherent apoptotic properties of LA. Overall, the collective findings on the effects of LA on UV irradiation appear to be controversial. Although there is evidence to suggest the beneficial effects of LA on photoprotection, the exposure of LA to UV has the potential to induce apoptosis and inflammation. Therefore, further examination is warranted.

### 4.5. Pro- and Anti-Cutaneous Inflammation

LA is an indirect precursor of PGE2, leukotriene and other inflammatory factors, and thus may be considered to have pro-inflammatory effects. However, recent studies in healthy adults have shown that increased dietary intake of LA does not elevate concentrations of inflammatory markers. Furthermore, epidemiological studies suggest that LA may be associated with reduced inflammation [66]. Several studies have investigated the anti-inflammatory effects of LA in both cultured cells and murine models. An in vitro study reported that LA treatment (18.75~150 μM) did not induce cytotoxicity and 25 µM of LA treatment did not induce the expression of IL-8 in keratinocytes [67]. Furthermore, LA exhibited an anti-inflammatory effect on *Cutibacterium acnes*-activated macrophages by suppressing the secretion of pro-inflammatory factors, including IL-1β, IL-6 and TNF-α [68]. In addition, the topical application of 1 mM LA has been shown to attenuate inflammation through the activation of PPAR-α in murine models of contact dermatitis [69].

Vegetable oils with high levels of LA such as sunflower oil (*Helianthus annuus* L.) have been employed in the treatment of inflammatory disorders [70]. A recent review of clinical studies on vegetable oils for dermal use indicated that oils high in LA and saturated fatty acids may have positive effects on inflammation-affected skin [71]. Moreover, the anti-inflammatory effects of LA-rich vegetable oils have been demonstrated. Bran extract of rice (Khao Dawk Mali 105), containing 31.62% LA, has been shown to reduce the production of nitric oxide (NO) in the RAW267.4 cell line [72]. Additionally, the topical application of LA-rich red ginseng oil (Panax ginseng Meyer) significantly inhibited UVC-induced cyclooxygenase-2 (COX-2) expression in the skin tissues of mice [65].

It is worthy to note that an increased ratio of ω-6 to ω-3 fatty acids in human diets may contribute to the development of inflammation [73]. The ω-3 PUFAs, eicosapentaenoic acid (EPA) and docosahexaenoic acid (DHA), are considered as anti-inflammatory. However, LA has been shown to inhibit the conversion from α-linolenic acid (ALA) to EPA, as they compete for the same elongases and desaturases involved in the biosynthesis of PUFAs [66,74]. Thus, a high intake of LA potentially creates a more inflammatory environment. Nevertheless, the interaction between ω-3 and ω-6 PUFAs in topical applications has not been investigated and requires further research.

A summary of studies conducted over the past decade examining the impact of diverse vegetable oils and extracts containing LA on skin health is presented in Table 1.

## 5. Functions of Linoleic Acid in Hair Growth

The hair follicle (HF) is the primary dermal appendage, exhibiting a complex and intricate structure with a cyclic growth pattern. The growth cycle of the hair follicle consists of three successive phases: the growth phase (anagen), the apoptosis-mediated degeneration phase (catagen) and the relatively quiescent resting phase (telogen). Activation of the typical WNT/β-catenin pathway is a prerequisite for the development and cycling of hair follicles. The issue of hair loss is becoming increasingly prevalent, necessitating the development of efficacious pharmaceuticals with minimal adverse effects. In recent years, there has been growing interest in the potential of vegetable oils and extracts to promote hair growth [76]. A summary of studies conducted over the past decade examining the impact of diverse vegetable oils and extracts containing LA on hair growth is presented in Table 2.

The effects of LA treatment on hair growth have been evaluated through biochemical assays, cultured cell models and animal models. The enzyme 5α-reductase catalyzes the conversion of testosterone into the more potent androgen, dihydrotestosterone (DHT), which is the primary pathogenic androgen in androgenic alopecia [76]. In vitro studies have demonstrated that LA can inhibit 5α-reductase derived from fresh rat livers with an IC_50_ of 130 ± 3 µmol [77]. Several studies have reported that the topical application of LA promoted hair growth in hair loss mouse models via the activation of WNT/β-catenin signaling and inhibition of the expression of Dickkopf-associated protein (DKK-1) [65,78,79]. Furthermore, the proliferation rate of dermal papilla cells (DPCs) in response to a treatment with 30 µg/mL LA was found to be significantly elevated. This effect is likely due to the increased expression of cell cycle proteins [80].

Various LA-rich vegetable extracts, including red ginseng oil, *Prunus mira* Köhne oil, rice bran extract and *Malva verticillate* L. seed extract, have been demonstrated to stimulate hair growth in vivo and in vitro. The topical treatment of red ginseng oil in testosterone-treated C57BL/6 mice significantly restored hair regenerative capacity, as evidenced by the induction of the anagen phase, the upregulation of the WNT/β-catenin and Shh/Gli pathways, the inhibition of transforming growth factor (TGF-β) and the enhancement of Bcl-2 [65,78]. The topical application of LA-rich *Prunus mira* Köhne oil to the skin of hairless C57BL/6 mice has been demonstrated to promote hair growth. This effect may be attributed to the regulation of the WNT/β-catenin pathway, which in turn facilitates the transition of hair follicles into the growth phase [79,81]. Rice bran extract, containing 38.42% LA, exhibited hair growth-promoting potential in C57BL/6 mice by upregulating the expression of vascular endothelial growth factor (VEGF), insulin-like growth factor-1 (IGF-1) and keratinocyte growth factor (KGF), while decreasing the expression of TGF-β [82]. Furthermore, the effects of KDML105 rice bran extract, containing 31.62% LA, on DPCs were assessed. Gene expression analysis revealed that rice bran extract treatment resulted in the downregulation of SRD5A2 (5α-reductase type 2) and TGF-β expression and upregulation of VEGF and CTNNB1 expression. These findings suggest the extract may play a role in anagen phase induction and angiogenesis in hair growth [72,83]. Additionally, the treatment of cultured DPCs with *M. verticillate* seed extract was found to result in increased WNT activity and elevated β-catenin levels. Furthermore, the LA-containing fraction of *M. verticillate* seed extract was found to promote cell proliferation and elevate β-catenin protein levels. Meanwhile, the extract was also observed to activate the expression of growth factors including IGF-1, KGF, VEGF and hepatocyte growth factor (HGF) [80,82].

It can be concluded that LA has the potential to regulate the expression of WNT/β-catenin, Shh/Gli and other crucial pathways associated with hair follicle growth. Moreover, the exogenous addition of LA has been observed to induce growth factors, including VEGF, IGF-1 and KGF, while simultaneously inhibiting TGF-β. This allows hair follicles to progress through the growth phase, regulate hair growth cycle and promote hair growth.

**Table 2:** Regulation of hair growth by vegetable oils or extracts containing linoleic acid.

Source of LA	Administration Type	Experimental Method	Effects	Refs.
Red ginseng oil (*Panax ginseng* Meyer)	Topical	10% red ginseng oil, 1% LA C57BL/6 mice treated with testosterone (TES) 28 days	Significant process of hair shaft growth; increased Bcl-2/Bax ratio; upregulation of WNT/β-catenin and recovery of Shh/Gli signaling pathways	[78]
Red ginseng oil (*Panax ginseng* Meyer)	Topical	50% red ginseng oil, 5% LA C57BL/6 mice 21 days	Increased follicle density and diameter; increased levels of β-catenin, p-GSK3β; increased levels of alkaline phosphatase (ALP), a biomarker of hair growth	[65]
*Prunus mira* Köhne	Topical	0.156 mg/cm^2^/d LA KM and C57BL/6 mice treated with sodium sulfide 6 days	Increased hair length and weight; increased mRNA and protein expression levels of β-catenin, GSK-3β, cyclin D1 and lef1	[79]
*Prunus mira* Köhne	Topical	15.06~60.26 mg/cm^2^/d KM and C57BL/6 mice 21 days	Increased number of hair follicles; promotion of hair follicles to enter the growth phase; increased expression of WNT 10b mRNA, β-catenin mRNA and protein and GSK-3β protein	[81]
Rice bran extract	Topical	38.42% LA C57BL/6 mice 4 weeks	Obvious hair growth; induction of hair follicles into the growth phase; increased number of hair follicles; increased mRNA expression levels of EGF, IGF-1 and KGF and decreased levels of TGF-β	[83]
*Malva verticillate* L. seed	Medium supplementation	3–100 µg/mL 3–30 µg/mL LA DPC 48 h	Increased expression of cyclin; promotion of growth factor expression; inhibition of DKK-1 expression; activation of the WNT/β-catenin signaling pathway; increased proliferation rate of DPC	[80]
*Malva verticillate* L. seed	Medium supplementation	10 µg/mL, 50 µg/mL DPC 24 h	Proliferation of DPC; increased β-catenin protein level; increased levels of IGF1, KGF, VEGF and HGF; enhanced phosphorylation of MAPKs (Akt and p38)	[82]
*Oryza sativa* L. cv. KDML105 Bran	Medium supplementation	0.1 mg/mL, 0.2 mg/mL DPC 24 h	Downregulation of SRD5A2 (5α-reductase type 2 gene) expression; promotion of β-catenin and VEGF expression and decreased expression of TGF-β1	[72]

## 6. Conclusions

As an essential fatty acid, LA in the skin can only be acquired from the diet or extracutaneous sites, given that it cannot be synthesized by the body. The biosynthesis of ω-6 PUFA from LA is limited in skin as the enzymes that convert LA to ω-6 PUFA are only expressed in differentiated sebaceous gland cells. Additionally, LA can be oxidized to produce oxylipins, including HODEs and oxo-ODEs, which function in cell proliferation, differentiation and the inflammatory processes. It is of particular importance to note that LA can be incorporated into ceramides, thereby playing a vital role in the formation of the skin barrier. Abnormalities in the metabolism of LA have been linked to the development of various skin diseases, including acne, atopic dermatitis and psoriasis. Studies have revealed that the topical application of LA or LA-rich vegetable oils exerts regulatory effects on skin health and hair growth (Figure 2). In the skin, the administration of LA has been demonstrated to confer a number of beneficial effects, including the repair of the skin barrier, the promotion of wound healing, skin whitening, photoprotection, and anti-inflammation. With regard to hair growth, exogenous LA has been demonstrated to exert an influence on the hair follicle growth cycle by inhibiting 5α-reductase activity, regulating the expression of critical signaling pathways related to hair follicle growth and inducing the release of growth factors.

In recent years, there has been growing consumer interest in natural, green, safe and healthy products. LA is predominantly found in natural vegetable oils, which has led to a greater acceptance among consumers for the application of LA-containing vegetable oils in cosmetics. Nevertheless, LA is a highly lipophilic molecule with a high partition coefficient, making it unsuitable for effective epidermal application. To address this, LA can be delivered via stable colloidal carrier systems, such as microemulsions (MEs) [84]. In addition, the thermal instability of LA can be improved by nanoencapsulating it with cyclodextrins [85].

However, a comprehensive understanding of the correlation between LA metabolism and skin diseases remains elusive. It remains unclear whether LA itself or its metabolites play a significant role in skin and hair growth, and whether LA’s effects vary across different skin types. Further research is necessary to elucidate the mechanisms through which LA functions in skin and hair growth and to gain insight into its interactions with other active ingredients. Such insights will aid the understanding and utilization of LA in the fields of cosmetics and pharmaceuticals.

## Figures and Tables

**Table 1:** Effects of linoleic acid or linoleic acid-rich vegetable oils on skin.

Efficacy	LA/Source of LA	Administration Type	Experimental Method	Effects	Refs.
Barrier function	Sunflower oil	Topical	167 mg/mL, Colworth–Wistar rats	Elevated TWEL levels in rats	[75]
Wound healing	LA	Topical	30 μM, Male BALB/c mice	Improved wound healing significantly after 48 h	[52]
Wound healing	LA	Topical	300 μL Male Wistar rats	Increased quality of wound healing tissue	[53]
		Medium supplementation	5, 25, 50, 100 and 200 mM, Neutrophils	Increased proportion of neutrophils; decreased thickness of the necrotic cell layer; increased VEGF-α and IL-1β	
Wound healing	LA	Medium supplementation	50, 100 µM, HaCaT	Increased cell migration, increased IL-8 expression and MMP-9 activity	[54]
Wound healing	Lucuma (*Pouteria lucuma* (Ruiz and Pav.) Kuntze) nut oil	Medium supplementation	20–100 μg/mL, zebrafish larva plate	Regeneration of zebrafish endothelial cells for faster wound closure	[55]
		Topical	500, 1000 μg CD-1 mice	New blood vessels in wounded areas in CD-1 mice	
Wound healing	Pumpkin seeds (*Cucurbita pepo* L.) oil	Topical	0.52 μL/mm^2^, Wistar rats	Shortened bleeding time; notable reduction in wound size and a remarkable capacity for healing	[56]
Skin whitening	LA	Medium supplementation	25 μM, B16F10 melanoma cells	Reduction of melanin levels by decreasing tyrosinase levels	[58]
Skin whitening, anti-aging	Spent coffee grounds oil	Medium supplementation	0.01 mg/mL, B16F10 melanoma cells and human skin fibroblasts	Inhibition of tyrosinase and related protein TRP-2 activity; inhibition of melanin production; inhibition of MMP-2 expression	[60]
Skin whitening, anti-oxidant activity	Rubber (*Hevea brasiliensis* (Willd. ex A. Juss.) Müll. Arg.) seed oil	Medium supplementation	0.0001–0.1 mg/mL B16-F10 melanoma cells and 3T3-L1 cells	Inhibition of tyrosinase and related protein TRP-2 activity; inhibition of melanin production; anti-oxidant activity	[61]
Skin whitening	LA	Topical	0.5% LA, UVB-stimulated hyperpigmented dorsal skin of brownish guinea pigs	Inhibition of melanin production in melanocytes; promotion of epidermal melanin desquamation	[59]
Photoprotection, anti-inflammatory	Red ginseng oil (*Panax ginseng* Meyer)	Topical	50% C57BL/6 mice treated with UVC	Inhibition of inflammation and apoptosis induced by UVC	[65]
Anti-oxidant activity, anti-inflammatory	KDML105 bran extract	Medium supplementation	0.1 mg/mL, RAW 264.7 macrophage cells	Scavenging ability of the DPPH radicals; decreased NO production in the RAW 267.4 cell line	[72]

## Abbreviations

Linoleic acid (LA); polyunsaturated fatty acid (PUFA); arachidonic acid (AA); γ-linolenic acid (GLA); dihomo-γ-linolenic acid (DGLA); α-linolenic acid (ALA); hydroxyoctadecadienoic acids (HODEs); oxo-octadecadienoic acids (oxo-ODEs); epoxy-9-keto-10-trans-octadecenoic acid (EKODEs); lipoxygenase (LOX); cyclooxygenase (COX); esterified-omega-hydroxy acyl-sphingosine (EOS); atopic dermatitis (AD); triglyceride (TG); corneocyte lipid envelope (CLE); tyrosinase (TYR); tyrosinase-related protein 1 (TRP1); hair follicle (HF); dermal papilla cells (DPCs).
